# Supplementary material for: Evidence the Isc iron–sulfur cluster biogenesis machinery is the source of iron for [NiFe]-cofactor biosynthesis in Escherichia coli
Source: Sci Rep. 2024 Feb 6;14:3026. doi: 10.1038/s41598-024-53745-2 (PMC10847431; doi:10.1038/s41598-024-53745-2)
Supplement: Supplementary file 1 — Supplementary Figures. [file 41598_2024_53745_MOESM1_ESM.pdf]

Supplementary Information

Evidence that the Isc Iron-Sulfur Cluster Biogenesis Machinery Delivers Iron for [NiFe]-Cofactor Biosynthesis in *Escherichia coli*

Alexander Haase[1‡], Christian Arlt[2‡], Andrea Sinz[2] and R. Gary Sawers[1*]

[1]Institute for Biology/ Microbiology, Martin-Luther University Halle-Wittenberg, Kurt-Mothes-Str. 3, 06120 Halle (Saale), Germany, [2]Institute of Pharmacy, Center for Structural Mass Spectrometry, Martin-Luther University Halle-Wittenberg, Kurt-Mothes-Str. 3a, 06120 Halle (Saale), Germany

* Send correspondence to: Gary Sawers, Institute for Biology/ Microbiology, Martin-Luther University Halle-Wittenberg, Kurt-Mothes-Str. 3, 06120 Halle (Saale), Germany. Tel: +49 345 5526350; Email: gary.sawers@mikrobiologie.uni-halle.de, Orcid: https://orcid.org/0000-0003-0862-2683

‡ These authors contributed equally to this work

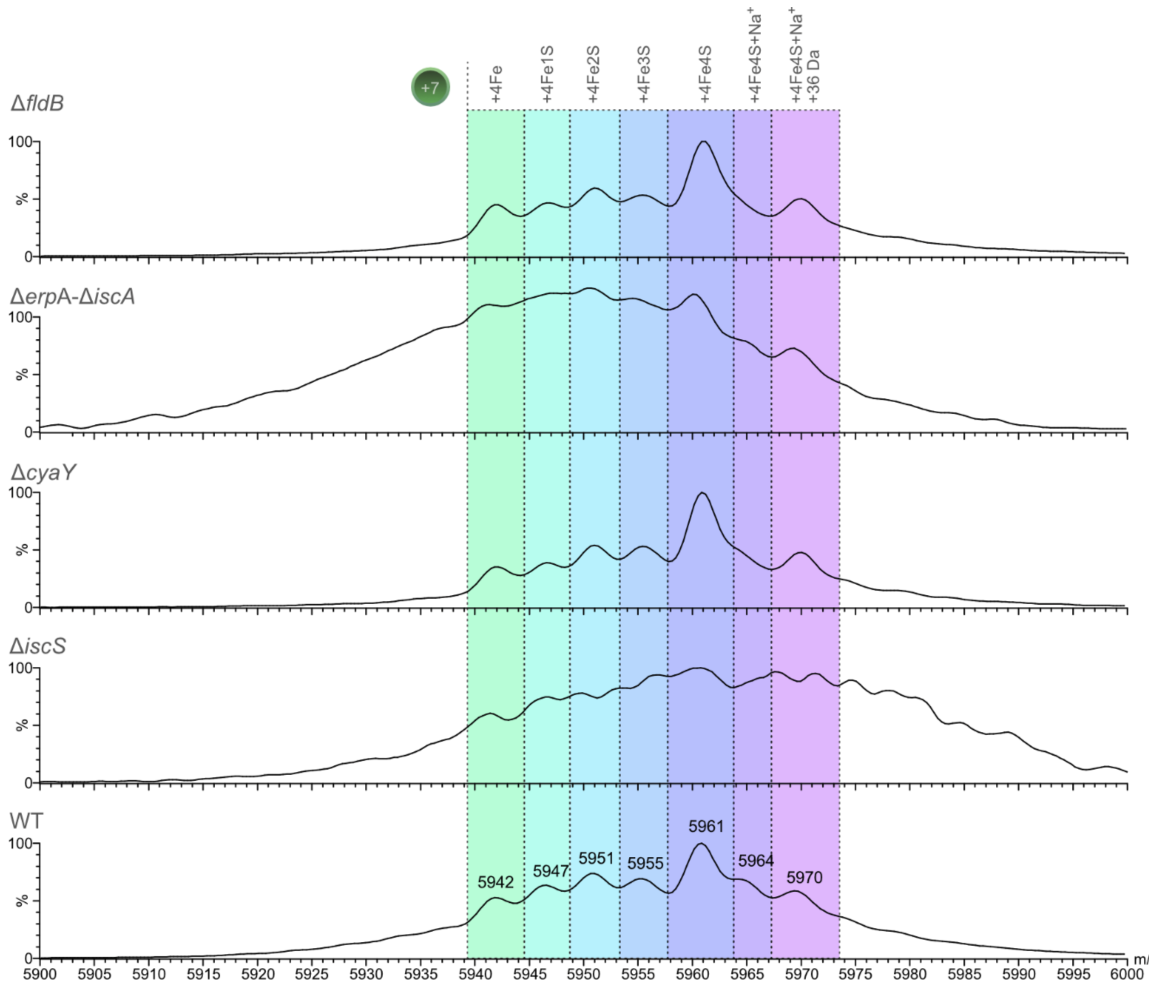

Figure S1. Native mass spectra of [4Fe-4S] cluster-containing HypD dissociated from HypCD complexes isolated from different iron metabolism mutants. Zoomed in native MS showing the +7 charged species of HypD including its [4Fe-4S] cluster dissociated from StrepII-tagged HypCD complex (charge state +12; collision energy 90V) isolated from strains MHD535 (Δ*cyaY*), PHB526 (Δ*iscS*), CP742 (Δ*iscA*-Δ*erpA*) and AS10 (Δ*fldB*) compared to the MC4100 (WT). All strains were transformed with plasmid pT-hypDCStrep.

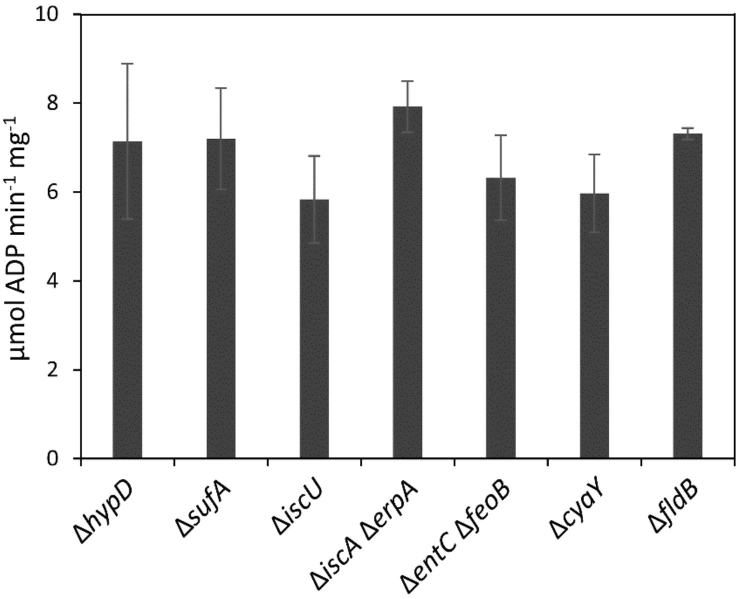

Figure S2. ATPase activity of HypCD complexes is unaffected by *isc* mutations. ATPase activity of purified, native HybG-HypD complexes isolated from the indicated mutants is shown. All strains carry plasmid pT-hypDCStrep encoding the StrepII-tagged HypCD complex.

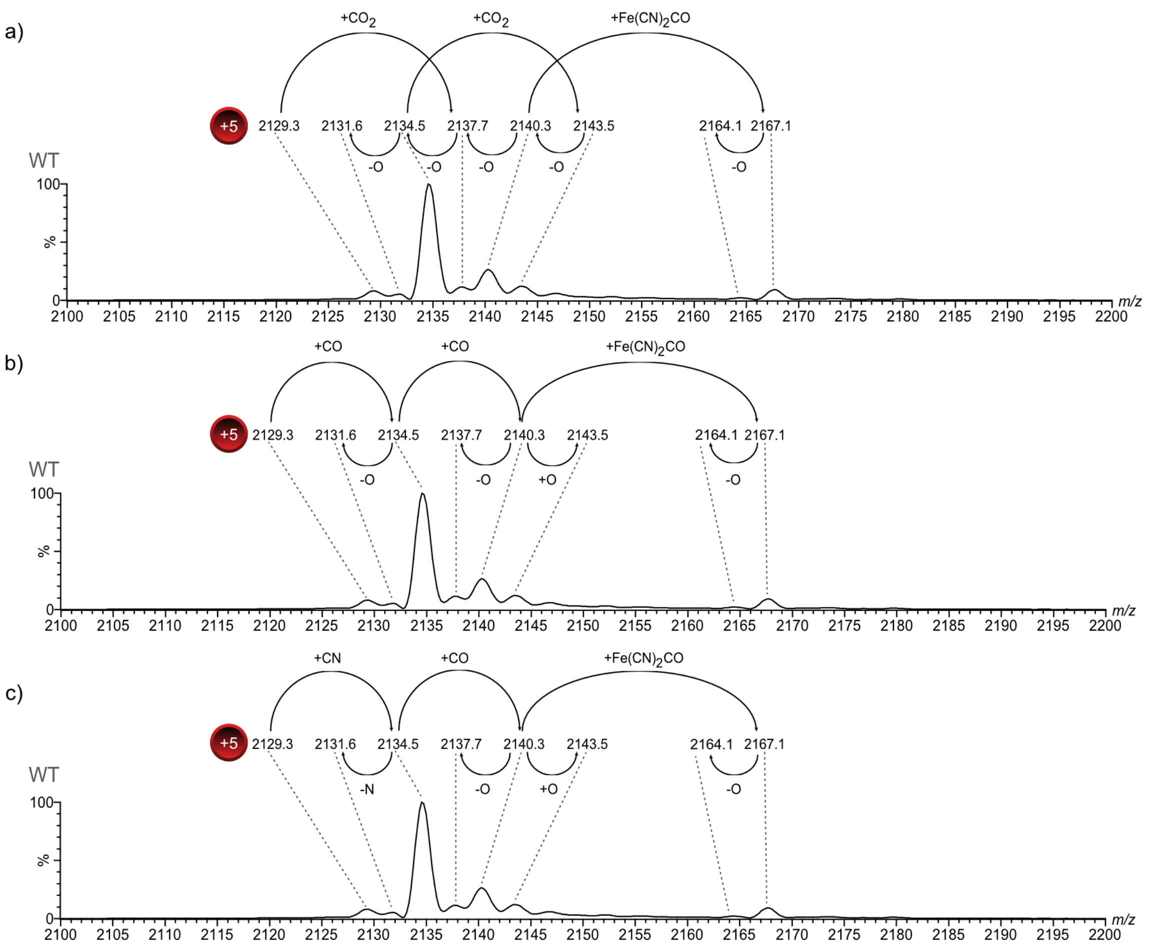

Figure S3. Overview of potential adducts on HypC. The three panels present different possible adducts accounting for the mass differences on HypC. – or +O signifies loss or addition of an oxygen. Note that the possibility of a methyl thiazolidine modification accounting for the +26 Da modification is not represented.

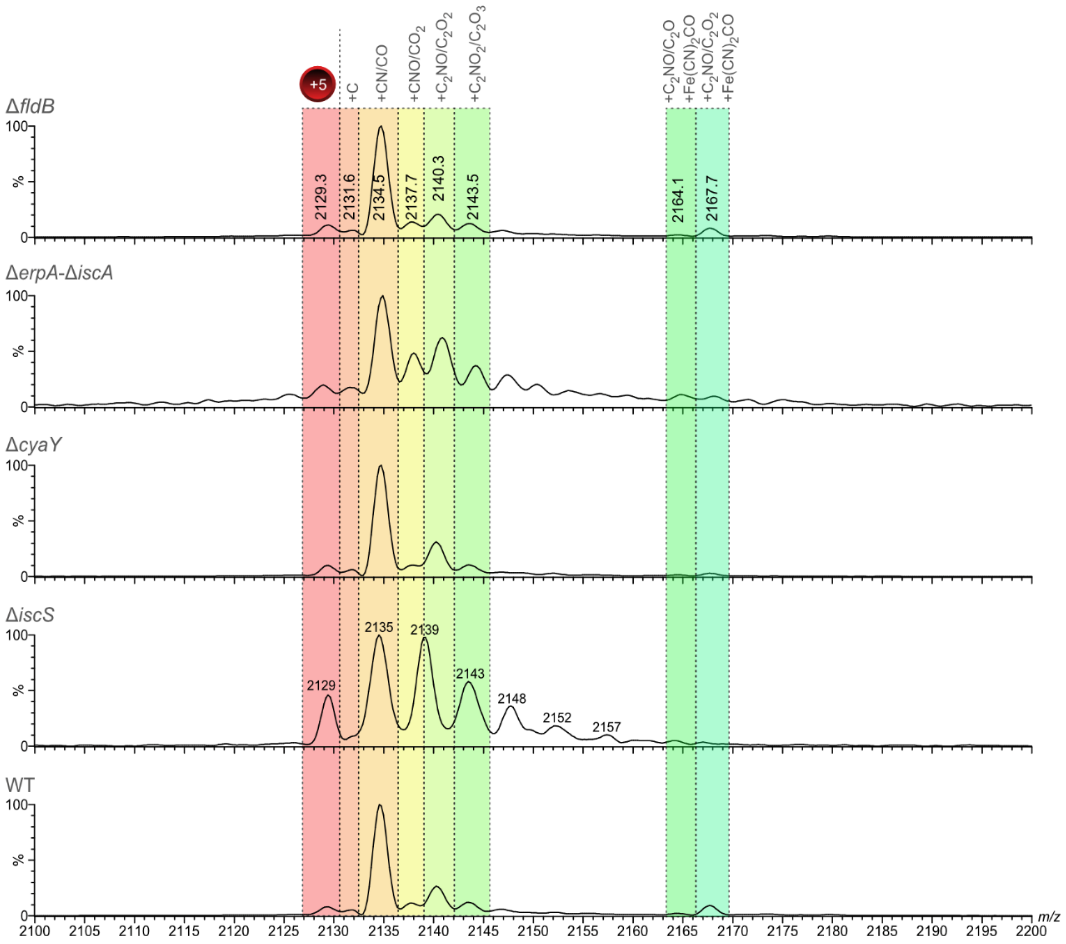

Figure S4. Native mass spectra of HypC dissociated from HypCD complexes isolated from different iron metabolism mutants. Zoomed in native MS showing the +5 charged species of HypC including its modifications dissociated from StrepII-tagged HypCD complex (charge state +12; collision energy 90V) isolated from strains MHD535 (Δ*cyaY*), PHB526 (Δ*iscS*), CP742 (Δ*iscA*-Δ*erpA*) and XYZ (Δ*fldB*) compared to the native HypCD complex (WT) isolated from DHP-D transformed with pT-hypDCStrep. Potential modifications are

indicated above the colored overlay. Note that a putative methyl thiazolidine modification accounting for the +26 Da species is not indicated in the Figure.

Original images used for generation of Figures 1 and 3 in the main manuscript

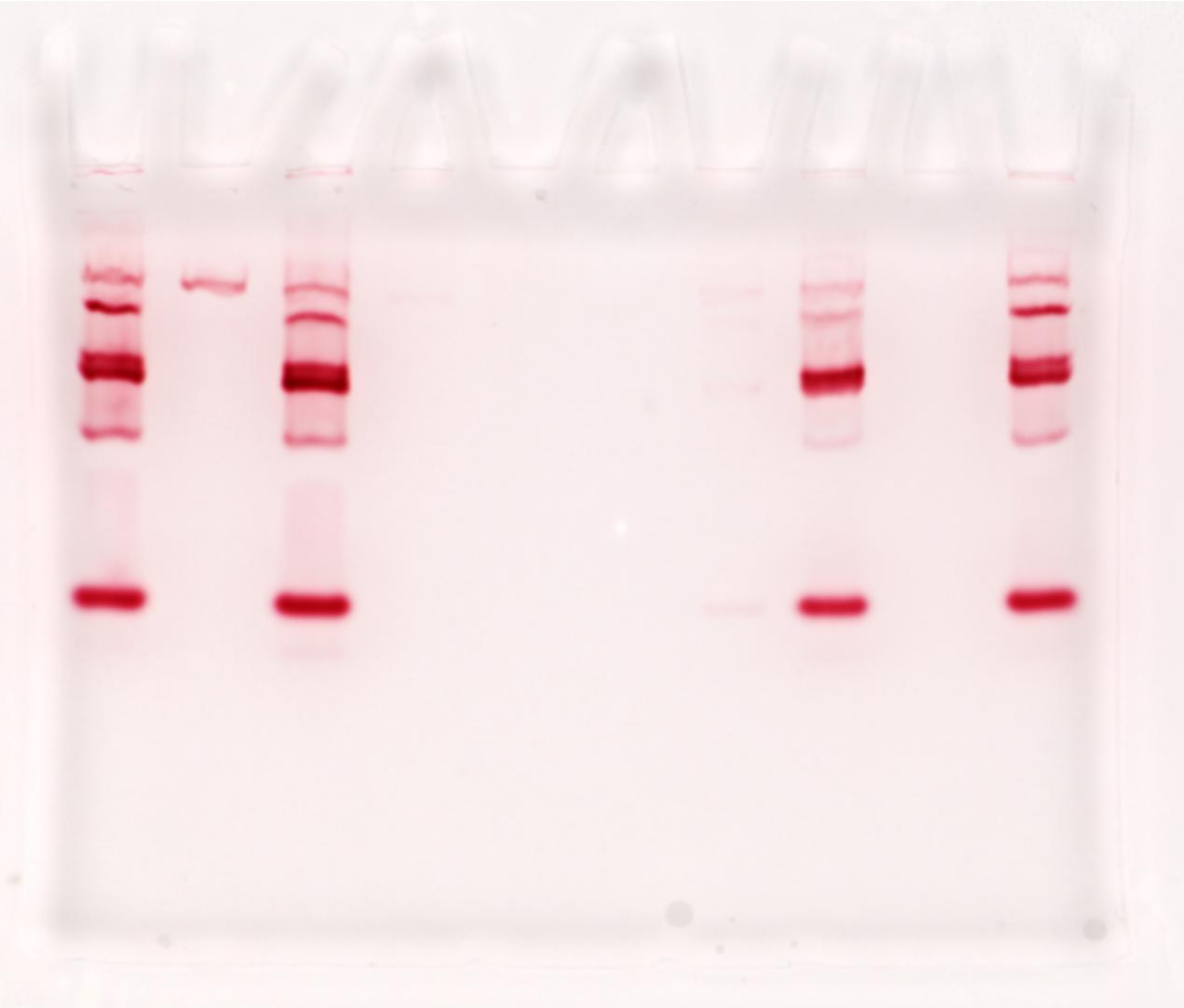

Image used to create Figure 1a.

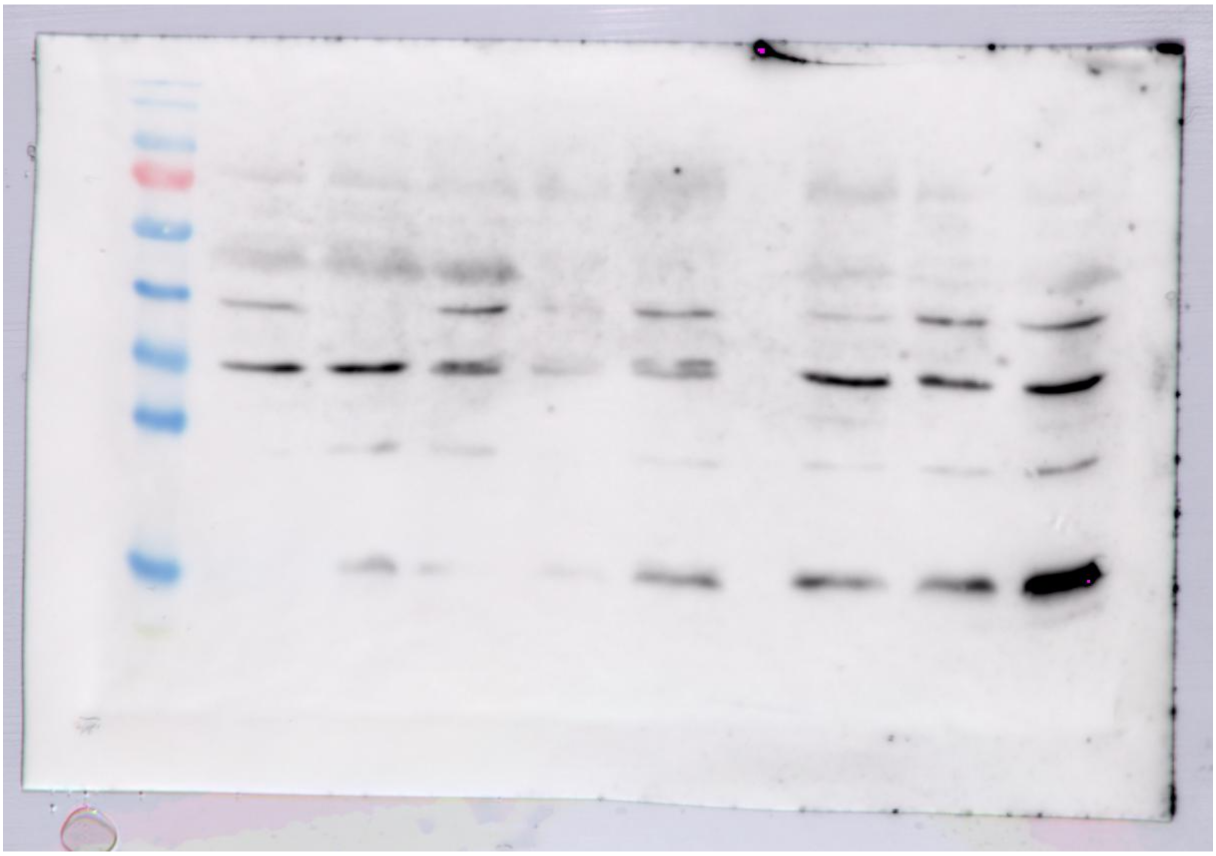

Western blot used to create Fig. 1b.

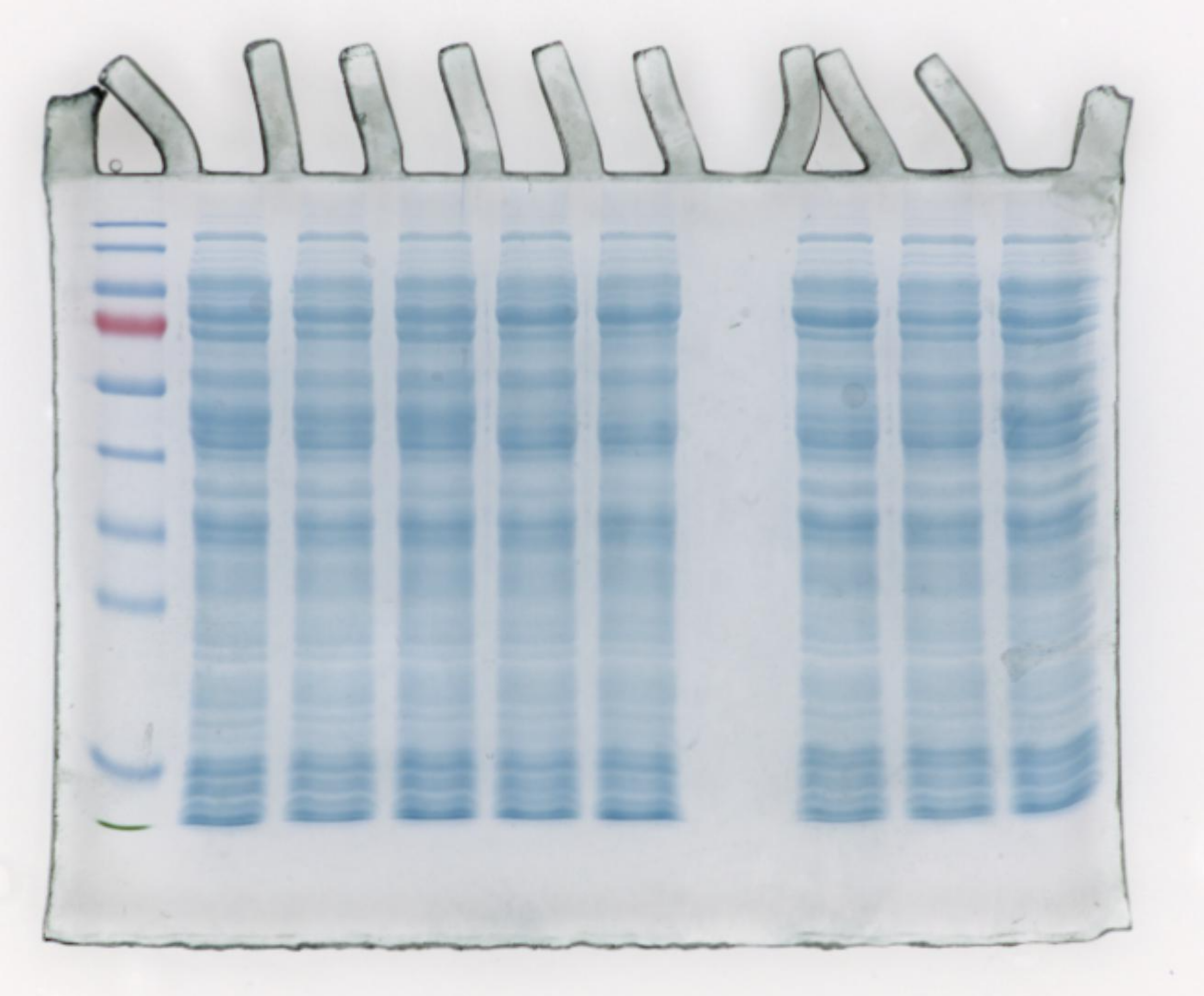

Image used to create Figure 1c.

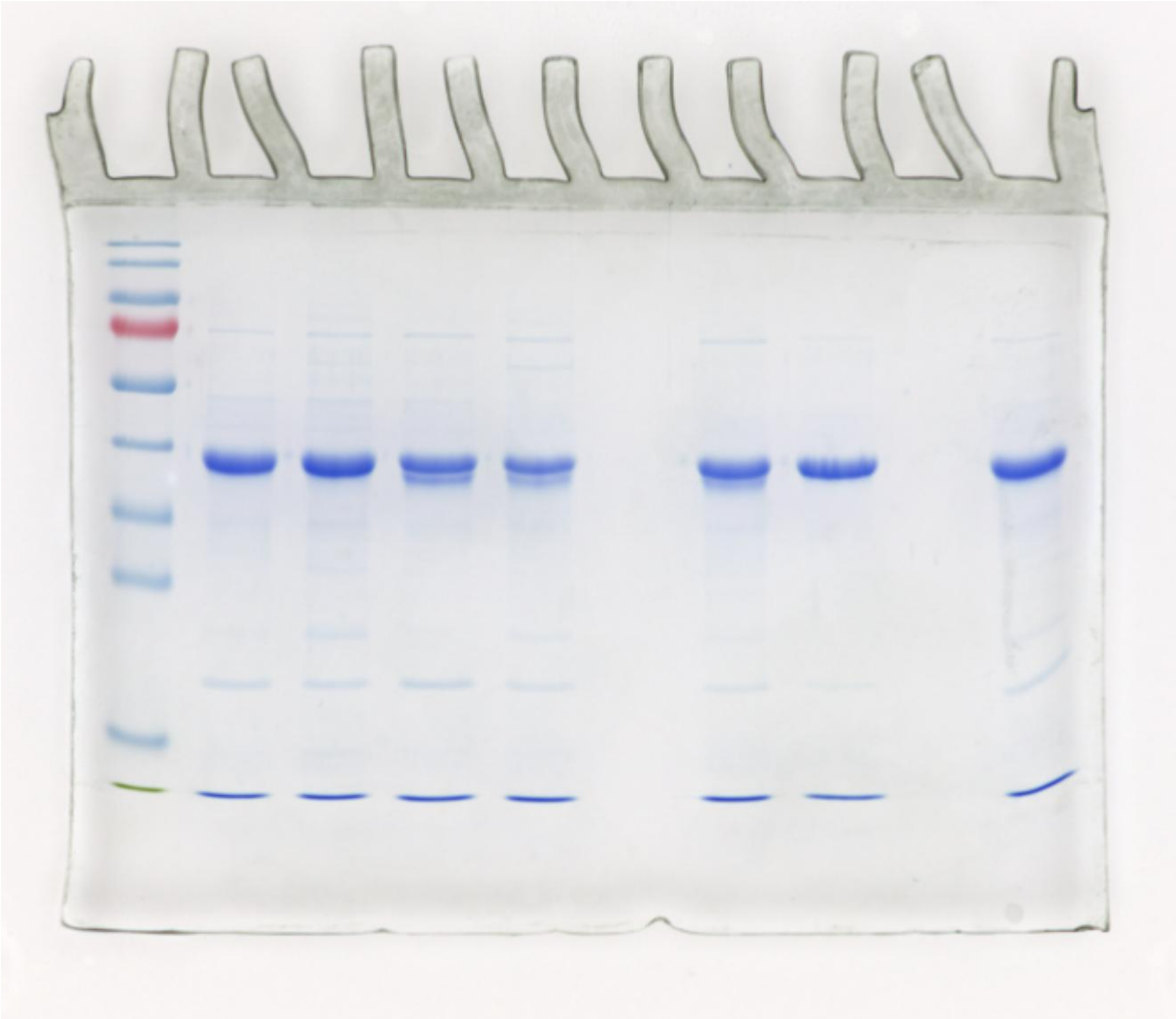

Image used to create Figure 3a.

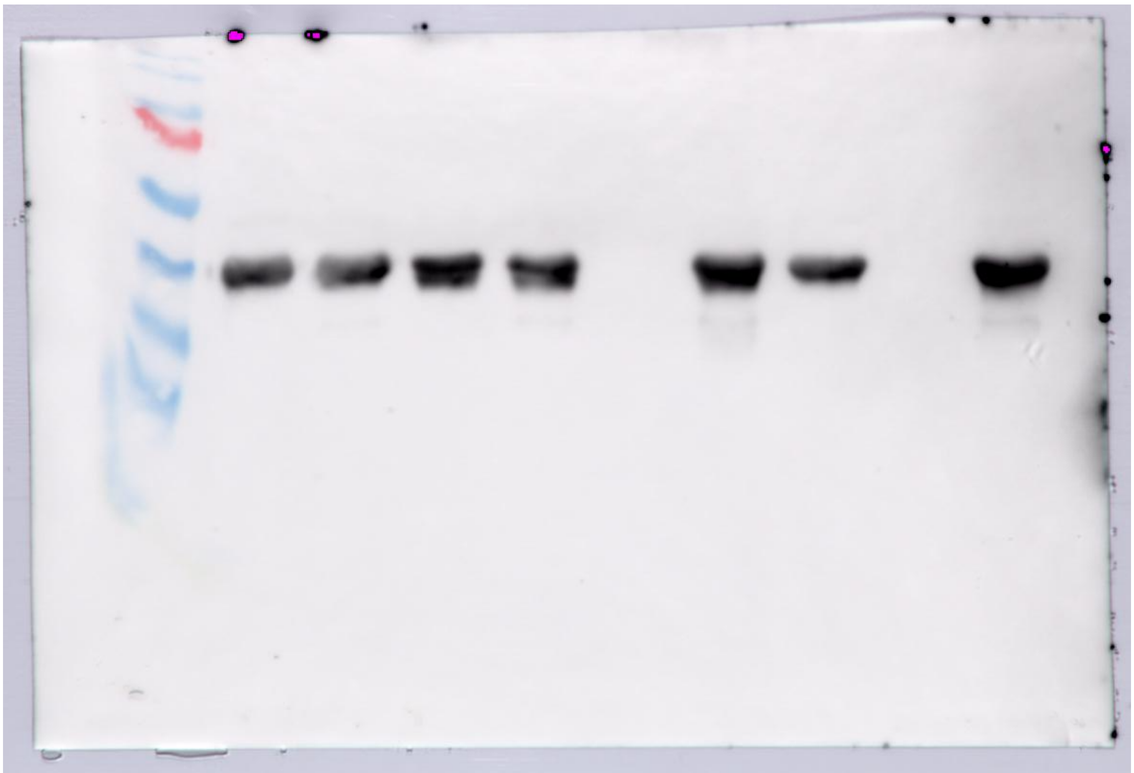

Image used to create Figure 3b.

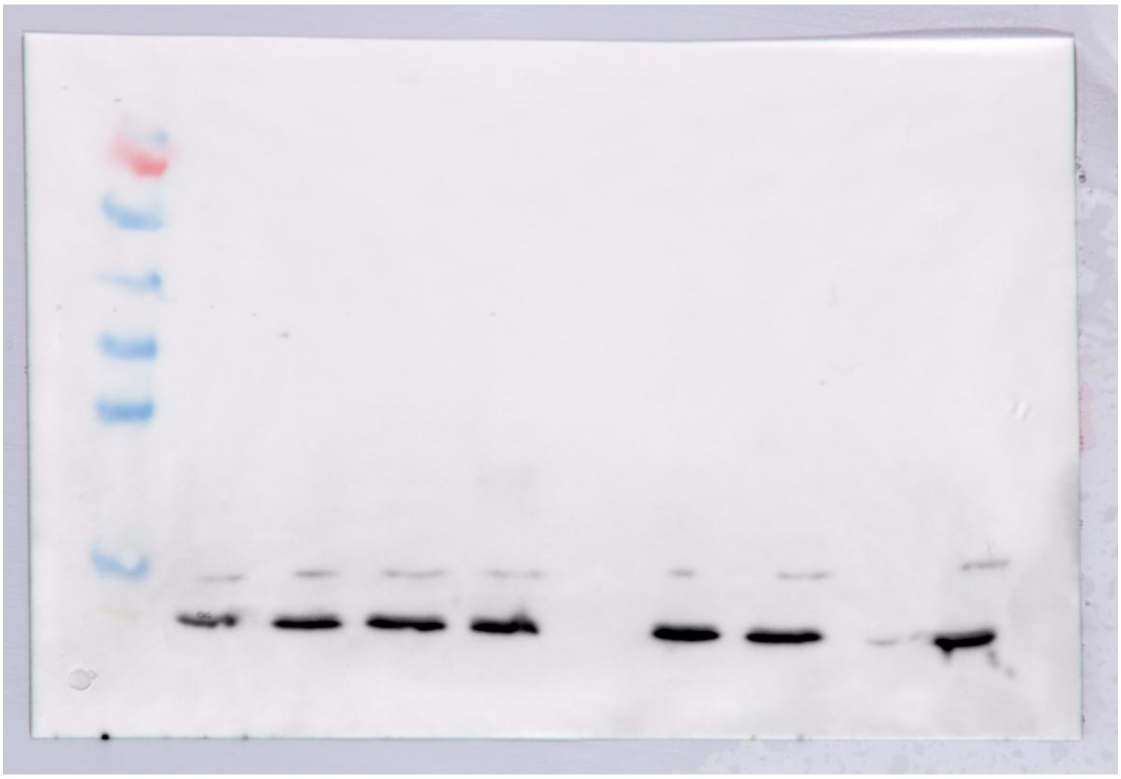

Image used to create Figure 3c.
